# The Expression of Selected Wnt Pathway Members (FZD6, AXIN2 and β-Catenin) in Canine Oral Squamous Cell Carcinoma and Acanthomatous Ameloblastoma

**DOI:** 10.3390/ani11061615

**Published:** 2021-05-29

**Authors:** Barbora Putnová, Iveta Putnová, Miša Škorič, Marcela Buchtová

**Affiliations:** 1 Laboratory of Molecular Morphogenesis, Institute of Animal Physiology and Genetics, Czech Academy of Sciences, 602 00 Brno, Czech Republic; bara.putnova@gmail.com (B.P.); putnovai@vfu.cz (I.P.); 2 Department of Pathological Morphology and Parasitology, University of Veterinary and Pharmaceutical Sciences, 612 42 Brno, Czech Republic; skoricm@vfu.cz; 3 Department of Anatomy, Histology and Embryology, University of Veterinary and Pharmaceutical Sciences, 612 42 Brno, Czech Republic

**Keywords:** squamous cell carcinoma, acanthomatous ameloblastoma, Wnt signaling, dog, oral tumor

## Abstract

**Simple Summary:**

The expression patterns of selected Wnt pathway molecules were analyzed in two of the most common canine oral neoplasia—canine oral squamous cell carcinoma (COSCC) and canine acanthomatous ameloblastoma (CAA). We found an overlap of areas with high expression of FZD6 and SOX2 in COSCC, while cytokeratin expression was low in these areas, indicating the low differentiation status of these cells. In CAA, FZD6-positive areas expressed cytokeratin and exhibited features of squamous metaplasia. Moreover, the expression of β-catenin and AXIN2 was higher in both CAA and COSCC than in the healthy canine oral epithelium. This work uncovered the distinct expression patterns of Wnt molecules in both lesions, indicating the involvement of this pathway in the pathology of canine oral cancers, which presents opportunities for their usage for the prediction of cell behavior or in the development of new therapeutic approaches.

**Abstract:**

The Wnt signaling pathway is well known to be involved in many types of human cancer; however, in veterinary medicine, the investigation of individual Wnt members’ expression, and their role in or association with oral tumor pathogenesis, is still underevaluated. We aim to determine the expression pattern of Frizzled-6 (FZD6) as one of the Wnt receptors in two of the most common canine oral neoplastic lesions—canine oral squamous cell carcinoma (COSCC) and canine acanthomatous ameloblastoma (CAA). While COSCC is a malignant tumor with aggressive biological behavior and a tendency to metastasize, CAA is a benign tumor with high local invasiveness. In CAA, the expression of FZD6 was mostly located in the center of the epithelial tumorous tissue, and cells exhibiting features of squamous metaplasia were strongly positive. In well-differentiated COSCC, FZD6 was expressed in the tumorous epithelium as well as the tumorous stroma. There was a negative correlation between cytokeratin expression and FZD6 expression in COSCC, where the central parts of the epithelial tumorous tissue were often FZD6-negative. The non-differentiated COSCC with low expression of cytokeratin exhibited a diffuse FZD6 signal. The invasive front with areas of tumor budding exhibited high FZD6 expression with a loss of cytokeratin expression. Moreover, the expression of β-catenin and AXIN2 was increased in comparison to gingiva. In conclusion, our study revealed significant differences in the expression patterns and the levels of FZD6 between COSCC and CAA, indicating the differential engagement of the Wnt pathway in these tumors.

## 1. Introduction

With the rapid developments in the field of veterinary oncology, there is an increasing need for a deeper understanding of the molecular regulations of animal cancer development. The oral cavity is one of the most frequent sites of neoplastic proliferation in dogs [1]. Here, we focus on two of the most common canine oral tumors of epithelial origin: oral squamous cell carcinoma and acanthomatous ameloblastoma [2].

Canine oral squamous cell carcinoma (COSCC) is the second most common malignancy in the oral cavity of dogs. Macroscopically, they usually form ulcerated masses and plaques on the tongue or buccal mucosa [3]. These lesions invade the surrounding structures, but the prognosis is auspicious after radical chirurgical excision at early stages [4]. The newest histologic classification of COSCC is based on a classification of human OSCC [5,6] and distinguishes conventional OSCC (which can be well differentiated, moderately differentiated and poorly differentiated), papillary OSCC, basaloid OSCC, spindle cell OSCC and adenosquamous OSCC [7]. Although the pathogenesis of this neoplasia is not yet fully understood, canine OSCC is considered to be a possible model for a human OSCC [8]. 

While COSCC exhibits numerous similarities to human OSCC, canine acanthomatous ameloblastoma (CAA) is unique to dogs. Moreover, ameloblastoma in humans is rare, but CAA is a frequent oral neoplasia in dogs [9,10]. Macroscopically, this tumor grows usually as a lobulated mass in the rostral area of the jaws [3,10]. The microscopic structure of this tumor typically involves prominent intercellular contacts and palisade cell arrangement in the outer epithelial layer [11]. CAA is a benign tumor; however, it can be locally invasive and the chirurgical excision has to be radical in order to prevent local recurrences [10,12]. CAA pathogenesis is poorly understood and two possible origins in this neoplasia have been discussed: from the odontogenous residual epithelium (similar to human ameloblastoma) [13] or from the oral epithelium [1]. CAA has also been shown to carry similar mutations as human ameloblastoma [14,15,16,17].

The wingless-related integration site pathway (Wnt) is crucial for tissue homeostasis, and its misregulation has been described in numerous diseases [18]. The Wnt/β-catenin pathway is one of the most studied signaling pathways and its dysregulation can lead to cancer development [19]. β-catenin acts as an effector in the canonical Wnt signaling pathway; it is involved in the regulation of downstream gene transcription and also in the cadherin-based cell–cell junctions. The loss of its expression or dislocation from the membrane to the cytoplasm is connected to a dysregulation of cell adhesion and brings higher risk for metastasis and poor prognosis in both canine and human OSCC [20,21,22,23]. If the Wnt canonical pathway is activated by the binding of Wnt ligands to FZD receptors, β-catenin is translocated into the nucleus, interacts with TCF/LEF proteins and converts them into transcriptional activators [24]. In the absence of a Wnt ligand, Wnt signaling is suppressed via the formation of a β-catenin destruction complex, in which AXIN2 is one of the key elements. The expression of AXIN2 is induced by Wnt signaling and AXIN2 has been suggested as a direct target of the Wnt pathway [24]. AXIN2 is also considered a tumor suppressor and mutations in the human gene coding this protein have been linked to colorectal carcinoma [25]. In the case of colorectal carcinoma, instead of acting as a tumor suppressor from a functional point of view, AXIN2 promotes the progression of the disease by promoting the epithelial–mesenchymal transition through the upregulation of SNAIL1 [26], and the overexpression of AXIN2 enhances the invasiveness of the colon carcinoma. AXIN2 gene was found to be overexpressed in meduloblastoma cell lines in which the Wnt signaling pathway was activated [27].

Here, we focus on the expression of transmembrane protein FZD6, which acts as a receptor of Wnt signaling. Expression of the gene coding *Fzd6* molecule was found to be higher in some human cancers, such as colorectal carcinoma, hepatocellular carcinoma, SCC and prostate cancer [28,29,30,31]; however, there is no information about its possible association with canine oral neoplastic lesions. We selected OSCC and CAA as the most common oral tumors in dogs and evaluated the expression of FZD6, as well as key Wnt target molecules such as AXIN2 and β-catenin, in these tumors.

## 2. Materials and Methods

### 2.1. Canine Tissues

In total, 30 tumors were immunohistochemically evaluated, out of which 15 were COSCC and 15 CAA. Normal canine gingiva was used as a control tissue (total number: 5). Samples were acquired from the archive of the Department of Pathological Morphology and Parasitology of the Veterinary and Pharmaceutical University Brno (Czech Republic) in the form of paraffin tissue blocks. All samples were obtained from dogs of private owners.

### 2.2. Immunohistochemical Analysis and Immunofluorescent Detection

Tissue sections of 5 µm thickness were prepared and stained with Hematoxylin–Eosin (HE). The alternative sections were used for immunohistochemical and immunofluorescent analysis. The following primary antibodies were applied for the detection of specific protein expression by immunohistochemistry (Table 1) or immunofluorescence (Table 2): β-Catenin PY 489, FZD6, pan-cytokeratin, Sex determining region Y box 2 (SOX2), Ki-67. In the case of immunohistochemistry, 3,3′-Diaminobenzidine (DAB) was used for visualization of the signal and Hematoxylin was used to counterstain the nuclei. For visualization of nuclei in the case of immunofluorescence, DRAQ5™ Fluorescent Probe Solution was used (Thermo Scientific™, Shanghai, China). Sections were photographed under bright-field illumination with a Leica compound microscope DMLB2 (Leica, Wetzlar, Germany) or SP8 Resonant Scanning Confocal microscope (Leica, Wetzlar, Germany) in the case of immunofluorescence.

## 3. Results

### 3.1. Expression of SOX2 and Ki-67 in COSCC and CAA of Dogs

**Figure 1:**
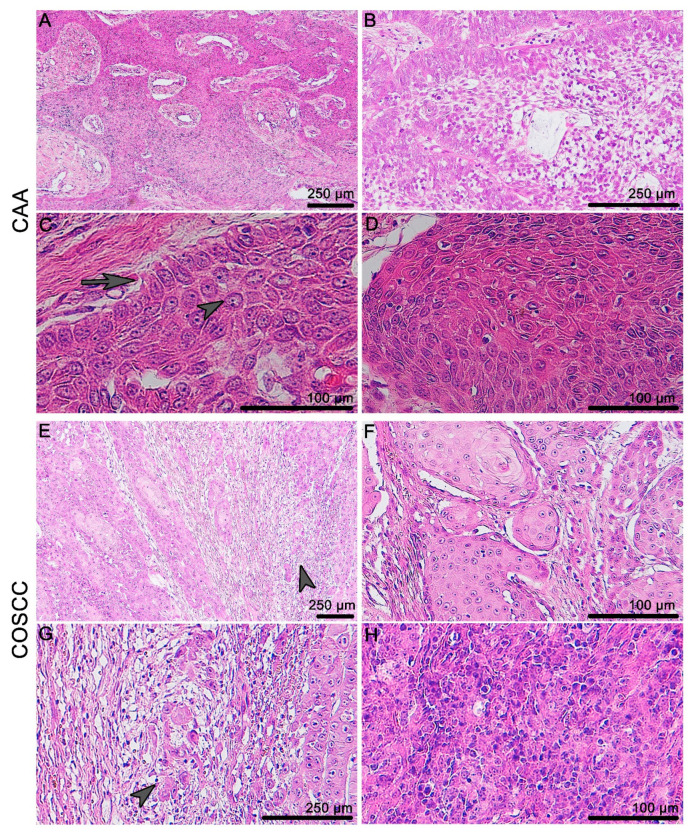
Microscopic anatomy of canine gingiva and histopathology of CAA and COSCC. (**A**) CAA grows invasively in the form of unencapsulated trabeculae of odontogenic epithelium surrounded by a primitive mesenchymal stroma. (**B**) The odontogenic epithelium can resemble stellate reticulum-like structures in the middle of the tumorous islets. (**C**) There is a characteristic palisading of the outer layer of epithelial cells, with often vacuolated cytoplasm, and the reverse polarity of nuclei (arrow) and prominent intracellular bridges can be found commonly in the centers of the islands (arrowhead). (**D**) Keratinization can occur as one of the features of this neoplasia. (**E**,**G**) Well-differentiated COSCC are characterized by an invasiveness and infiltrative “budding” on the periphery (arrowhead). (**F**) The level of keratinization is linked to the level of differentiation. (**H**) Poorly differentiated (non-keratinizing) tumors display marked cellular atypia and pleomorphism.

COSCC and CAA exhibit distinct morphological features and the diagnosis is usually performed based on common histological staining. CAA usually grows invasively in the form of odontogenic epithelial trabeculae, which are surrounded by mesenchymal stroma (Figure 1A–D). In contrast, well-differentiated COSCC are characterized by an invasiveness and infiltrative “budding” of epithelial protrusions on the tumorous tissue periphery (Figure 1E–H).

**Figure 2:**
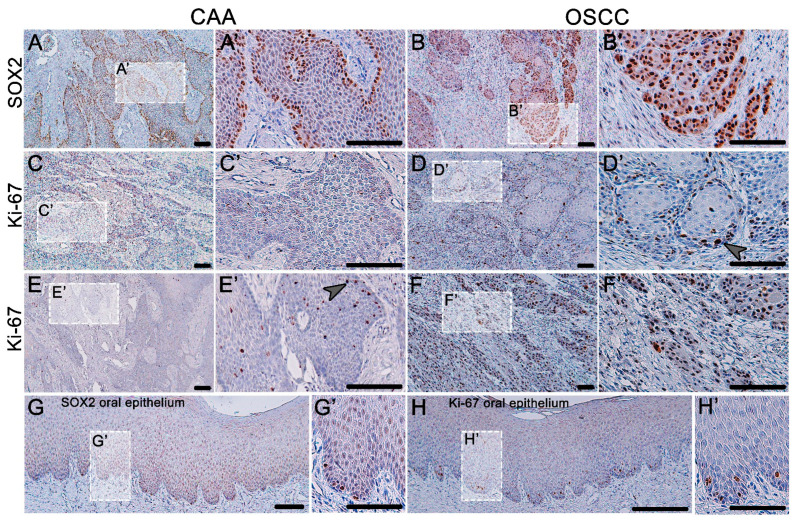
Expression of SOX2 and Ki-67 in CAA and COSCC. (**A**) In CAA, the expression of SOX2 is strong and is almost exclusively localized in the outer layer of neoplastic epithelial tissue. (**B**) In COSCC, the expression of SOX2 is strong and diffuse (**B**’) with an especially distinct signal in the areas of the tumor-invasive front and tumor budding. (**C**,**E**) The proliferation activity (Ki-67-positive cells) is rarely present in CAA (**E**’); only occasionally, there are areas of positive cellular nests (arrowhead). (**D**) In well-differentiated COSCC, the Ki-67-positive cells are present mainly in the outer layers of the epithelial islets (arrowhead). (**F**) Poorly differentiated COSCCs are diffusely Ki-67-positive with strong expression. (**G**) Normal canine oral epithelium has scattered SOX2-positive cells along the basement membrane and Ki-67 exhibit the same dispersed expression pattern (**H**). (**A’**–**H’**) Higher magnification insert.

We performed analyses of the transcription factor SOX2’s expression and determination of the proliferation activity in these tumors (Figure 2). In the normal canine oral epithelium, the most SOX2-positive cells were located in the basal layer while the expression faded in the superficial direction through the epithelial layers. Compared to the state in the normal gingiva, where the SOX2 cells were located only sparsely on the basal layer of the oral epithelium, there was strong expression of SOX2 in both COSCC and CAA. In CAA, the expression was mainly distributed in the outer layer of the epithelial cells. There was strong positivity in the areas of COSCC “budding” and in the invasive front area.

Ki-67 protein was used to determine the areas of cell proliferation (Figure 2). In normal gingiva, the Ki-67 cells were scattered only in the basal layer. In the case of CAA, the proliferating cells were distributed randomly in the middle areas of the tumorous epithelium; thus, the expression of SOX2 and Ki-67 was not colocalized in this tumor. In the case of well-differentiated COSCC, the proliferating cells were localized around the outer layer of the epithelial nests, resembling the expression stratification of the normal epithelium. In cases of a poorly differentiated COSCC, there was strong, diffuse Ki-67 expression.

### 3.2. Expression of FZD6 in CAA and COSCC

**Figure 3:**
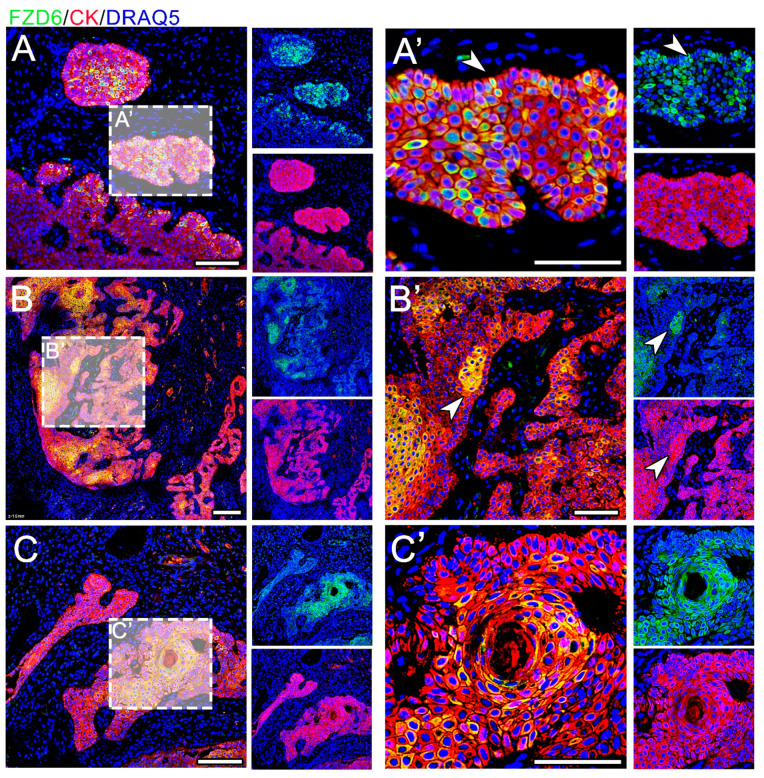
Expression of FZD6 in CAA. (**A**–**C**) The expression of FZD6 is strong and located in the cytoplasm and membranes. (**B,B’**) The signal is mostly located in the center of epithelial tumorous tissue (arrowhead) (**A**’) but some scattered positive cells are located in the palisading outer layer of the tumorous tissue (arrowhead). (**C,C’**) FZD6 expression is very strong in the areas of squamous metaplasia. Scale bar = 100 μm.

**Figure 4:**
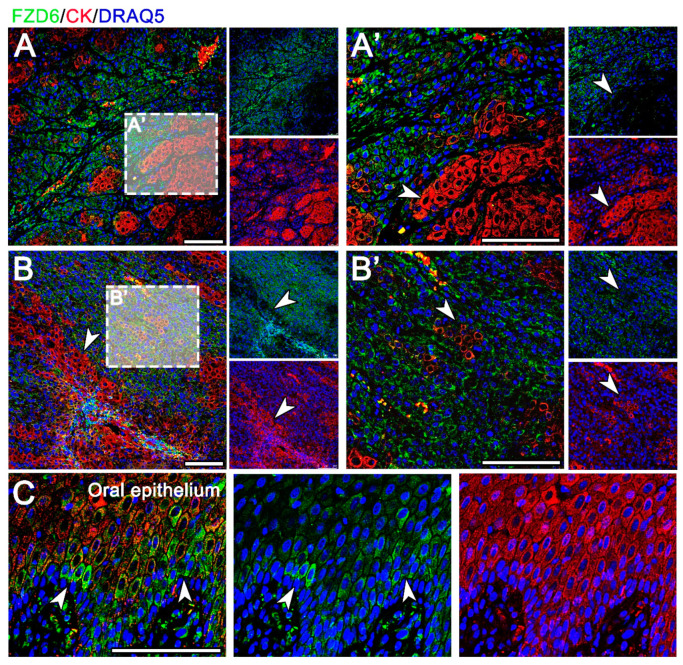
Expression of FZD6 in COSCC. (**A**) In a well-differentiated OSCC, FZD6 is expressed in the neoplastic epithelium and in the tumorous stroma. (**B**) In poorly differentiated OSCC with low expression of cytokeratin, the FZD6 signal is diffusely dispersed. (**A**’,**B**’) There is a distinct negative correlation between cytokeratin signal and FZD6 expression in OSCC (arrowheads). (**A**’) The invasive front with areas of tumor budding demonstrates high FZD6 expression with a loss of cytokeratin expression. (**C**) Normal canine oral epithelium displays scattered cytoplasmic positivity mostly in the basal layer (arrowheads). Scale bar = 100 μm.

The expression of FZD6 was found in both analyzed tumors—CAA and COSCC—and the expression pattern of FZD6 varied significantly between these two lesions (Figure 3 and Figure 4). The co-expression of FZD6 and cytokeratin was investigated to correlate FZD6 expression with the differentiation status of neoplastic cells.

In CAA, FZD6 expression was located in the membranes and cytoplasm of epithelial cells (Figure 3). FZD6-positive cells were located in the center of epithelial tumorous tissue; however, a signal was located in the palisade-like outer layer of the tumorous tissue. Moreover, FZD6 expression was very strong in the areas of squamous metaplasia.

In the well-differentiated OSCC, FZD6-positive cells were located in the neoplastic epithelium and in the tumorous stroma (Figure 4A). The poorly differentiated areas of OSCC, exhibiting low expression of cytokeratin, were diffusely positive for FZD6 (Figure 4). There was a distinct negative correlation between the intensity of cytokeratin and FZD6 expression in OSCC (Figure 4). Areas of tumor budding in the invasive front displayed high FZD6 expression with a loss of cytokeratin expression (Figure 4). In contrast, scattered cytoplasmic positivity of FZD6 was observed in the basal layer of the normal oral epithelium (Figure 4C).

### 3.3. Expression of β-Catenin and AXIN2 in CAA and COSCC

**Figure 5:**
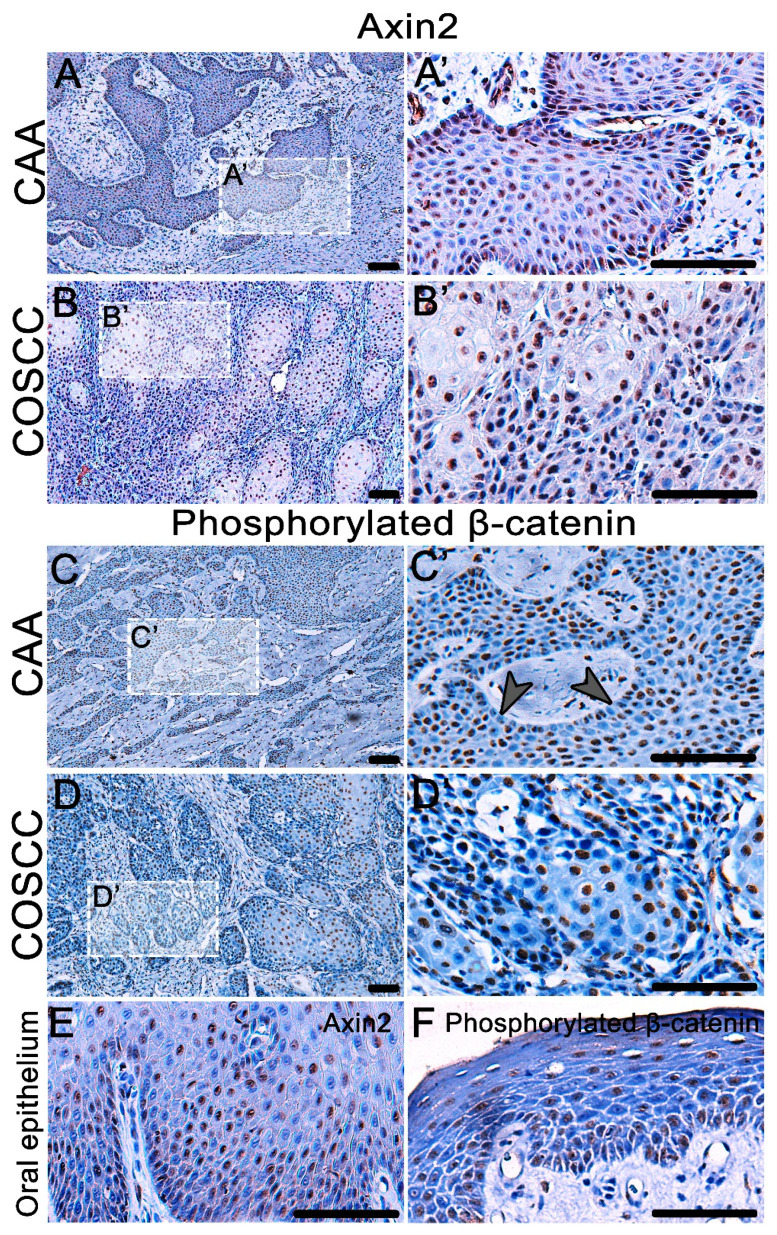
Detection of AXIN2 expression and β-catenin phosphorylated at Tyr 489 in COSCC and CAA. (**A,A´, B,B´**) AXIN2 is overexpressed in the nuclei of both CAA and COCC. (**E**) The expression of AXIN2 in physiological canine oral epithelium is weak. (**C,D**) There is a strong nuclear expression of phosphorylated β-catenin in CAA and COSCC. (**C,C´**) In CAA, the signal is often weakened in the outer layer of the epithelial tumorous islets (arrowheads). (**D,D´**) There is a strong nuclear expression of phosphorylated β-catenin in COSCC, the signal is stronger in the larger and more differentiated cells in the middle of tumorous islets. (**F**) In the normal canine oral epithelium, the expression of phosphorylated β-catenin is very weak. Scale bar = 100 μm. (A’–D’) Higher magnification insert.

As we observed areas of increased expression of FZD6 in both types of tumors, we sought to uncover whether downstream molecules of Wnt signaling displayed upregulation in the analyzed areas. We selected two downstream targets—phosphorylated β-catenin (phospho PY489) and AXIN2. The expression of both markers was higher in both CAA and COSCC than in the healthy canine oral epithelium (Figure 5E,F).

AXIN2 was expressed in numerous nuclei of both CAA and COCC (Figure 5A,B). There was also a strong nuclear expression of phosphorylated β-catenin in CAA and COSCC (Figure 5C,D). In CAA, less β-catenin-positive cells were located in the outer layer of the epithelial tumorous islets. While a strong nuclear expression of phosphorylated β-catenin was found in COSCC, the β-catenin signal was stronger in the larger and more differentiated cells in the middle of tumorous islets. In the physiological canine oral epithelium, the expression of phosphorylated β-catenin was very weak (Figure 5F).

## 4. Discussion

The biological behavior of CAA and COSCC varies, especially considering the speed of growth and their local invasiveness [10]. Slow growth of CAA corresponds to the low numbers of Ki-67-positive cells found in this neoplasm, which is in contrast to COSCC, with high Ki-67 expression. The Ki-67 labeling index is considered to be a prognostic factor of many canine cancers [32,33,34,35], and, in COSCC, it was significantly associated with a lymph node metastasis [36]. Ki-67 expression might be also a negative prognostic marker for human patients with OSCC [37]. The distribution of the Ki-67-positive cells in CAA and COSCC has not been evaluated yet. In well-differentiated COSCC, the Ki-67-positive cells are located mostly in the areas of tumor budding and outer layers of the tumorous islets. This phenomenon corresponds to the invasive tumor front, where the Ki-67 expression was described to be high in human OSCC [38]. Similarly, in breast cancer tissue, the Ki-67-positive cells also tend to be located in the outer layers of the tumor nest rather than in the center [39]. There is no close counterpart in the human pathology to the CAA. CAA partially resembles human central and peripheral ameloblastoma, but there are some distinguishable features. There are four types of human ameloblastoma: conventional, peripheral and unicystic. By its macroscopic appearance, CAA resembles human peripheral ameloblastoma (HPA); however, HPA does not involve bone. Due to its bone invasion, CAA resembles human conventional ameloblastoma, of which the acanthomatous histological type is the most similar [40]. In this study, the Ki-67 proliferation rate was low, with positive cells encountered both to peripheral and central areas, similar to human ameloblastoma [41,42].

To illustrate the differences regarding the growth potential, we further performed analyses of the expression of transcription factor SOX2 and determination of cells with progenitor potential in CAA and COSCC, taking into consideration also expression in the normal gingiva as a reference structure. SOX2 expression has been suggested to act as a prognostic factor in various cancers in humans, and its increased expression has been associated with the malignancy and metastatic spread of tumors [43,44,45]. In oral lesions, SOX2 expression has been described in human odontogenic keratocysts and ameloblastoma [46]. In comparison to human ameloblastomas, where SOX2 expression is low, CAA exhibits higher expression of SOX2. Human odontogenic keratocysts are usually strongly SOX2-positive, resembling the expression in CAA. The SOX2-positive progenitor cells were mainly present in large numbers in the outer layer of the tumorous islets. This differential expression through individual layers was similar to the normal gingiva, where SOX2-positive cells were located in the basal layers. The high expression of SOX2 in the COSCC invasive front and tumor “budding” corresponds to its aggressive biological behavior, but the increased presence of SOX2-positive cells in outer layers of the CAA epithelium is surprising and further investigation of the fate of these cells is needed.

In the case of human oral cancer (OSCC), Wnt signaling components are often overexpressed [47,48]. In veterinary medicine, research on the Wnt pathway has, up to now, mostly concentrated on canine mammary tumors and canine cutaneous melanoma [49,50,51], while oral tumors have not been evaluated. Here, we selected for further analyses the Wnt receptor (FZD6) and also downstream molecules (AXIN2, β-catenin) to determine potential differences in Wnt pathway activity in CAA and COSCC. 

In both analyzed lesions, high expression of FZD6 was observed. In the case of COSCC, the areas with strong FZD6 expression were also strongly SOX2-positive, indicating high numbers of progenitor cells in these areas. Similarly, alterations of several FZD receptors have been in many human cancers and FZD6 has been found to be increased in human liver, prostate, colorectal cancer and cutaneous SCC [28,29,30,31]. FZD6 is considered a potential cancer stem cell marker in human neuroblastoma and high expression is linked to a poor prognosis [52]. Ablation of FZD6 expression in human mammary cancer cell lines was found to inhibit cell invasion, lead to a more symmetrical growth pattern and inhibit the metastatic potential of tumorous cells [53]. Enforced expression of SOX2 leads to the inhibition of Wnt/β-catenin signaling activity [54]. In the veterinary literature, however, there is not yet evidence of FZD6 expression and its association with tumor progenitor and proliferation status, to the best of our knowledge. Considering the similar distribution of SOX2- and FZD6-positive cells in COSCC, the FZD6-positive tumors might demonstrate higher growth status and thus more aggressive biological behavior [53]. Additional experimental data are, however, needed to confirm the correlation between the level of FZD6 expression, SOX2 positivity and the prognosis of COSCC patients. 

In COSCC, we noticed an association among the tumor differentiation of cell keratinization and FZD6 expression. Areas of weak cytokeratin expression displayed the highest FZD6 signal. The low cytokeratin expression in FZD6-positive areas of COSCC indicates that these areas correspond to the sites with low differentiation status. In CAA, squamous differentiation (metaplasia) is a fairly rare event [11]. In CAA with squamous metaplasia, we noticed exactly the opposite pattern to that in COSCC—the areas of strong cytokeratin expression exhibited high FZD6 expression. Abnormally increased expression of FZD6 in poorly differentiated cells of COSCC as well as well-differentiated areas of CAA suggest differences in the malfunctioning of the Wnt pathway in these tumors and variability in the cell response, where the alteration of Wnt signaling results in a completely different fate of neoplastic cells.

Next, we also wanted to analyze downstream components of the Wnt pathway in CAA and COSCC with the aim of uncovering whether FZD6 expression can be correlated with the alteration of downstream signaling and whether there are differences between these two oral lesions. We noticed overexpression of AXIN2 in both CAA as well as COSCC when compared to a normal gingiva. In human ameloblastoma, there was also strong immunostaining of AXIN2 in comparison to a normal oral mucosa [55], and high expression levels of AXIN2 in human SCC were associated with tumor size and recurrence [56]. Moreover, increased levels of AXIN2 were highly correlated with the malignant transformation of an oral leukoplakia in humans [57], indicating possibly similar associations in canine oral tumors.

β-catenin is a wide-ranging molecule in terms of its function. In the cell adhesion junctions, it acts as a link between α-catenin and cadherins [58,59]. The loss of β-catenin expression is therefore linked to the disruption of these cell contacts and it is associated with tumor spread—namely, metastasis initiation. Moreover, it is a well-studied prognostic factor used mainly in human SCC [60,61,62,63,64]. In COSCC, the loss of this adhesion molecule was also connected to the epithelial–mesenchymal transition in the invasive front of this tumor [21]. In terms of Wnt pathway activity and its involvement in the tumorigenesis of CAA and COSCC, the phosphorylated form of β-catenin expressed in the nucleus seems to be pivotal and there are no data in the literature about the expression of this form in canine cancers. During the activation of the Wnt pathway, β-catenin is phosphorylated at tyrosine 489 (PY489 β-catenin) and it is transferred to the nucleus, resulting in the presence of “nuclear” or “active” β-catenin, where it functions, among others, as an activator of oncogenic targets [65].

Activation of the canonical Wnt pathway seems to be dissimilar in these two canine oral neoplasia. In COSCC, the nuclear β-catenin expression was found to be strong and located focally mostly in the areas of well-differentiated cells and in the zones with low SOX2 expression. In contrast, the nuclear β-catenin expression was often lost in areas of SOX2-positive cells in the outer palisading layer of CAA. Because β-catenin is translocated to the nucleus in the “Wnt-on” state [66], we suggest that the well-differentiated cells in COSCC are those where Wnt signaling is activated, whereas in the less differentiated SOX2-positive (progenitor) cells, the Wnt signaling is disrupted, which might lead to the aggressive behavior of these cells.

## 5. Conclusions

Expression of FZD6, β-catenin and AXIN2 indicates the activation of the Wnt signaling pathway in both CAA and COSCC. Their expression pattern correlates with the progenitor marker SOX2 and the proliferation status of tumorous epithelial cells; however, the distribution of positive cells is distinct in these two neoplasms. Our results indicate the distinct involvement of Wnt signaling in these two oral pathogeneses in dogs.

## Figures and Tables

**Table 1:** List of primary and secondary antibodies used for immunohistochemical analysis.

Primary Antibody	Company	Catalog No.	Pre-Treatment	Detection	Dilution	Secondary Antibody
SOX2	Cell Signaling Technology	2748	Citrate buffer (pH 6.0), 20 min, 98 °C in water bath	IH-P (DAB), Hematoxylin	1:100	VECTASTAIN ABC HRP Kit (Peroxidase, Rabbit IgG), Vector Laboratories, USA, cat. No. PK-4010
AXIN2	Abcam	32197	Citrate buffer (pH 6.0), 20 min, 98 °C in water bath	IH-P (DAB), Hematoxylin	1:200	VECTASTAIN ABC HRP Kit (Peroxidase, Rabbit IgG), Vector Laboratories, USA, cat. No. PK-4010
β-Catenin PY 489	DSHB	10144551	Citrate buffer (pH 6.0), 20 min, 98 °C in water bath	IH-P (DAB), Hematoxylin	1:50	VECTASTAIN ABC HRP Kit (Peroxidase, Mouse IgG), Vector Laboratories, USA, cat. No. PK-4002
Ki-67	Cell Marque	275-R-16	Citrate buffer (pH 6.0), 20 min, 98 °C in water bath	IH-P (DAB), Hematoxylin	1:200	UltraView Universal DAB Detection Kit, Ventana, cat. No. 05269806001

**Table 2:** List of primary and secondary antibodies used for immunofluorescence detection.

Primary Antibody	Company	Catalog No.	Pre-Treatment	Detection	Dilution	Secondary Antibody
FZD6	Assay Biotechnology	G260	Citrate buffer (pH 6.0), 20 min, 98 °C in water bath	IF (Alexa Fluor 488), DRAQ5	1:100	Goat anti-Rabbit IgG (H+L) Cross-Adsorbed Secondary Antibody, Alexa Fluor 488, Thermo Fisher Scientific, USA, cat. No. A11008
pan Cytokeratin	Abcam	961	Citrate buffer (pH 6.0), 20 min, 98 °C in water bath	IF (Alexa Fluor 568), DRAQ5	1:1	Goat anti-Mouse IgG (H+L) Cross-Adsorbed Secondary Antibody, Alexa Fluor 568; Thermo Fisher Scientific, USA, cat. No. A11004

## Data Availability

Raw data are available on request.
